# Effects of Angiotensin-Converting Enzyme Inhibitor Derived from *Tropaeolum majus* L. in Rat Preimplantation Embryos: Evidence for the Dehydroepiandrosterone and Estradiol Role

**DOI:** 10.1155/2014/209207

**Published:** 2014-03-20

**Authors:** Emerson Luiz Botelho Lourenço, Juliane Centeno Muller, Ana Claudia Boareto, Caroline Gomes, Ana Carolina Lourenço, Rhanany Alan Calloi Palozi, Thiago Bruno Lima Prando, Arquimedes Gasparotto Junior, Paulo Roberto Dalsenter

**Affiliations:** ^1^Instituto de Ciências Biológicas, Médicas e da Saúde, Universidade Paranaense, P.O. Box 224, 87502-210 Umuarama, PR, Brazil; ^2^Departamento de Farmacologia, Universidade Federal do Paraná, P.O. Box 19031, 81531-990 Curitiba, PR, Brazil; ^3^Laboratório de Farmacologia e Toxicologia de Produtos Naturais, Universidade Paranaense, P.O. Box 224, 87502-210 Umuarama, PR, Brazil

## Abstract

Although several studies have shown the inhibitory effects of *Tropaeolum majus* extracts (HETM) on angiotensin-converting enzyme (ACE) activity, no studies have been carried out during the beginning of pregnancy, when humoral and hormonal imbalance may affect zygote and early embryo transport. This study investigates whether HETM can affect embryonic development when administered during the one-cell-blastocyst period. Pregnant Wistar rats received orally the HETM (3, 30, and 300 mg/kg/day) from the 1st to the 7th gestational day. Rats were killed on the 8th day of pregnancy and the following parameters were evaluated: clinical symptoms of toxicity (including organ weights), number of corpora lutea, implants per group, preimplantation losses ratio, and the serum levels of dehydroepiandrosterone (DHEA), estradiol, and progesterone. No clinical symptoms of maternal toxicity were evidenced. On the 8th day of pregnancy, the levels of DHEA and estradiol were increased and significant preimplantation losses were observed at all doses used. The present study reveals that the HETM can raise levels of DHEA and estradiol and induce difficulty in the embryo implantation in the early stages of pregnancy. The data contributes significantly to the safety aspects of using this natural product when trying to get pregnant or during pregnancy.

## 1. Introduction

*Tropaeolum majus* L. (Tropaeolaceae) is a native plant of the Andes in South America and it is widely distributed around the world. In Brazil, it is popularly known as chaguinha, capuchinha, or nastúrcio and it is used as diuretic and antihypertensive [1]. Furthermore, a booklet recently published by the Empresa Brasileira de Pesquisa Agropecuária (EMBRAPA) recommends the cultivation of this species as a source of vitamin C and minerals to be used as complementary and alternative medicine [2]. Due to its nutritional properties, this species has also been used in pregnancy, birth, and postpartum care in many rural areas in Brazil.

Recently, we have shown the efficacy of the hydroethanolic extract obtained from* Tropaeolum majus* (HETM) leaves as diuretic and antihypertensive, relating its effects to the flavonoid isoquercitrin, which was able to inhibit the angiotensin-converting enzyme and release bradykinin and prostaglandins [3–6]. Even though several studies proved its therapeutic effects, toxicological tests are also necessary to ensure the safe use of these compounds. Worldwide several regulatory agencies are responsible for the regulation of these substances and possess specific legislations for evaluation of efficacy and safety (such as Organization for Economic Co-operation Development (OECD)). Studies of preclinical toxicology are demanded in Brazil by Agência Nacional de Vigilância Sanitária (ANVISA) especially for the register of phytotherapic products [7].

Few toxicological studies on* Tropaeolum majus* can be found in the literature. Gasparotto Junior et al. [3] and Gomes et al. [8] reported lack of toxicity after acute and subchronic administration of HETM in rats. Likewise, HETM was unable to produce (anti)estrogenic or (anti)androgenic activities in the short-term in vivo screening assays performed [9]. Although the primary data of the literature described the relative safety of using* Tropaeolum majus* in both males and in females, no studies on HETM have been carried out during the beginning of pregnancy, when humoral and hormonal imbalance, as well as the action of some drugs, may affect zygote and early embryo transport. Hence, this study was designed in order to verify whether HETM affects embryonic development when administered to pregnant rats during the one-cell-blastocyst period, which comprehends the phases of tubal transit and implantation.

## 2. Materials and Methods

The methods used in this study were submitted to and approved by the Institutional Ethics Committee, Federal University of Parana, PR, Brazil, under Protocol number 383, following the international ethics principles for animal experimentation. The experimental design comprises part of the ICH protocol for the analysis of embryonic development, stage B [10, 11].

### 2.1. Experimental Model

Wistar rats were obtained from the Federal University of Parana, Brazil, and maintained under controlled conditions at 22 ± 2°C and a constant 12 h/12 h light/dark cycle. Three-month-old nulliparous rats weighing from 160 to 180 g were used in this study. The male rats were previously mated to attest fertility. The day of sperm detection in the vaginal smear was considered day 0 of pregnancy, and these females were randomly separated into the treatment and control groups and were housed individually in polypropylene cages (414 × 344 × 168 mm). Standard food pellets (Nuvital, Curitiba, PR, Brazil) and tap water were available* ad libitum*.

### 2.2. Plant Material, Preparation, and Dosage of the* Tropaeolum majus* Extract (HETM)

The leaves of* T. majus* were collected at the botanical garden of Universidade Paranaense (UNIPAR) (Umuarama, Brazil), which is located at 430 m of altitude above sea level (S23°47′55–W53°18′48). The plant was identified by Dr. Mariza Barion Romagnolo (UNIPAR, Brazil). A voucher specimen is deposited at the Herbarium of this university under number 2230.

The leaves were air-dried in an oven at 40°C for 4 days and then cut and pulverized. The resulting dried powdered plant material was macerated for 7 days in 90% ethanol solution. The solvent was removed using a rotary vacuum evaporator under reduced pressure and lyophilized, giving up 15.39% of the dry material extracted (HETM). The chemical composition of HETM was previously described [5].

The HETM doses were prepared according to the higher therapeutic dose capable of inducing diuresis and hypotension [3–5], including two lower doses as a safety factor. Doses of 3, 30, and 300 mg/kg of HETM were administrated once a day in 5 mL/kg of aqueous solution, through gavage, from the 1st to the 7th day of pregnancy.

### 2.3. Experimental Groups

Animals were randomly distributed into four experimental groups of 10–15 animals each: control (aqueous solution 5 mL/kg/day), HETM 3 (*Tropaeolum majus* 3 mg/kg/day), HETM 30 (*Tropaeolum majus* 30 mg/kg/day), and HETM 300 (*Tropaeolum majus* 300 mg/kg/day).

### 2.4. Experimental Protocol

At first, in order to evaluate the HETM toxicity on the maternal organism, the pregnant rats were observed during a 60-minute period after being treated with this substance. This procedure aimed to identify clinical symptoms of toxicity, such as piloerection, behavioral alterations (hyper- or hypoactivity, head flicking), tremors, convulsion, and death. Furthermore, food and water intake were monitored, once a day, and body weight gain or loss was monitored every two days [10, 12].

The pregnant rats were euthanized by decapitation and autopsied on the 8th day of pregnancy. The liver, kidneys, adrenal glands, uterus, and ovaries were dissected and weighed. Organ weights are reported as absolute and relative weights.

The uterine horns were sectioned longitudinally and the number of implants was determined for the calculation of (1) the implants per group ratio (number of implants/number of corpora lutea) × 100 and (2) the preimplantation losses per group (100 − implantation rate) [10].

Additionally, blood samples were collected and dehydroepiandrosterone (DHEA), estradiol, and progesterone levels were measured in serum using a microparticle enzyme immunoassay (AxSYM Estradiol and Progesterone assay). Kits were purchased from Abbott Laboratories (Abbott Park, IL, USA).

### 2.5. Statistical Analysis

The data obtained were processed through variance analysis (ANOVA), followed by Bonferroni's test, in those cases in which samples were normal and homoscedastic. For the data without homoscedastic samples and normal distribution, the nonparametric Kruskal-Wallis test was used, followed by the Mann-Whitney test. Chi-square test was used for the analysis of the preimplantation losses per group rate. Differences were considered significant at a probability level of 5% (*P* < 0.05).

## 3. Results

### 3.1. Maternal Toxicity

No clinical symptoms for maternal toxicity (head flicking, hyper- or hypoactivity, piloerection, tremors, convulsions, or deaths) were observed in any of the experimental groups. Moreover, the estimated food and water intake did not present significant differences between the control and treated groups (data no shown). Also, there were no significant changes in body weight as well as in the absolute and relative weights of liver, kidneys, adrenals, ovaries, and uterus (Table 1).

### 3.2. HETM Treatment Induces Increase of DHEA and Estradiol Levels in Pregnant Wistar Rats

**Figure 1:**
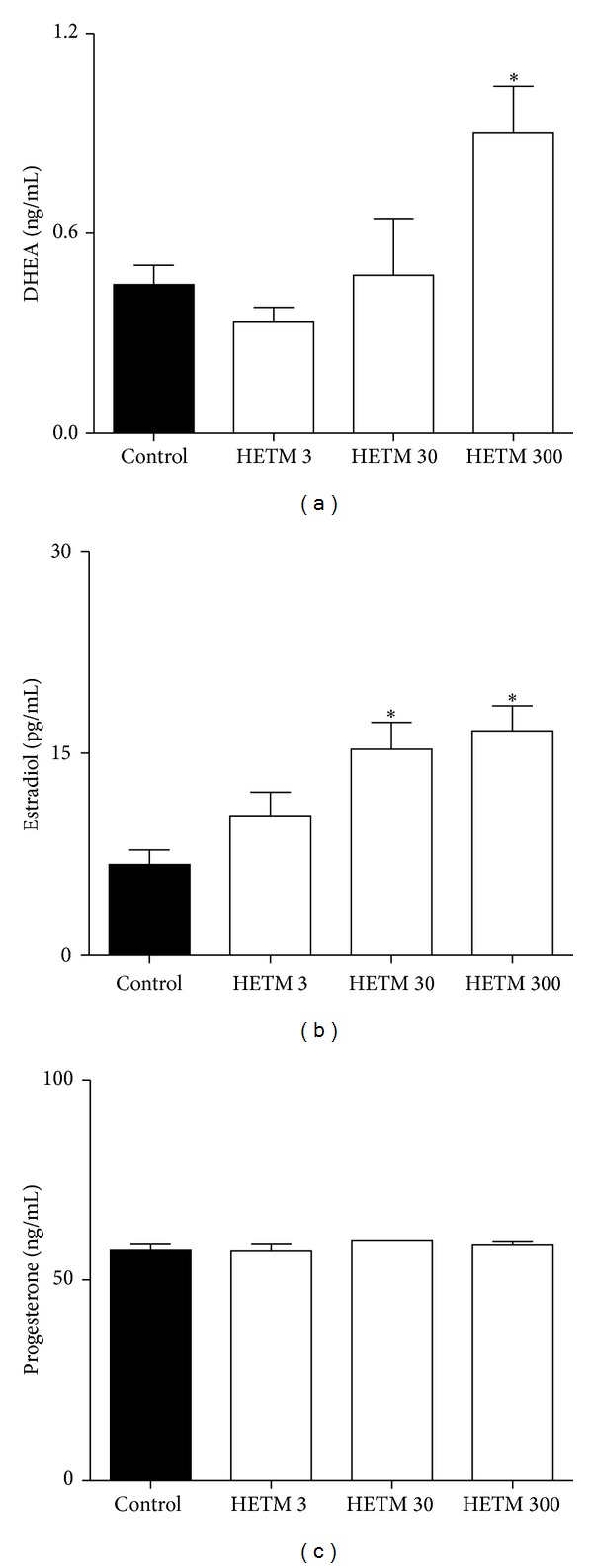
Serum levels of dehydroepiandrosterone (DHEA) (a), estradiol (b), and progesterone (c) of female Wistar rats treated orally with HETM (3, 30, and 300 mg/kg) or control (aqueous solution) from the 1st to the 7th day of pregnancy. Blood samples were obtained on the 8th day of pregnancy. Each bar represents the mean ± S.E.M. of the group. *The significance levels in comparison to control groups (one-way ANOVA followed by Bonferroni's test); *P < 0.05.

The daily treatment with HETM (300 mg/kg) significantly increased the serum DHEA (control: 0.41 ± 0.06; HETM 300: 0.90 ± 0.14 ng/mL; *P* < 0.05) as well as the estradiol levels when compared to the control group (control: 6.73 ± 1.08; HETM 30: 15.31 ± 1.99; HETM 300: 16.67 ± 1.84 pg/mL; *P* < 0.05). Progesterone levels were not affected by any treatment (Figure 1).

### 3.3. HETM Treatment Induces Preimplantation Losses in Wistar Rats Treated from Day 1 to 7 of Pregnancy

**Figure 2:**
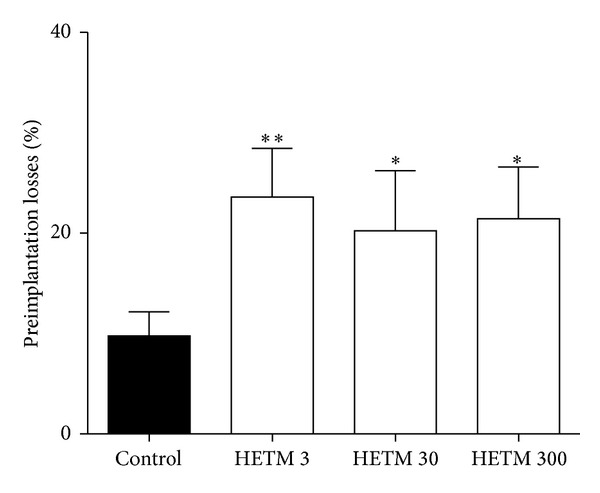
Percentage of preimplantation losses in relation to the total number of corpora lutea in female Wistar rats treated orally with HETM (3, 30, and 300 mg/kg) or control (aqueous solution) from the 1st to the 7th day of pregnancy. The graph shows the preimplantation losses in uterine horns on the 8th day of pregnancy. Each bar represents the mean ± S.E.M. of the group. *The significance levels in comparison to control group (Chi-square test); **P < 0.01 and *P < 0.05.

Figure 2 shows the percentage of preimplantation losses in female Wistar rats after treatment with HETM. The values obtained for doses of 3, 30, and 300 mg/kg were, respectively, 23.6 ± 4.85, 20.2 ± 5.99, and 21.4 ± 5.15% of losses. The control group had ~9% of preimplantation losses.

## 4. Discussion

Historically, women have used herbal drugs in pregnancy to treat pregnancy related illnesses and for their own health and well-being. Herbal drugs are often promoted as “natural” and “safe” alternatives to conventional drugs. These claims may especially appeal to pregnant women who are often concerned about their unborn child's well-being. Nevertheless, in several countries, herbal drugs are not currently subject to the same regulations as conventional drugs, and hence there is little or no testing of purity, safety, or teratogenicity. Still, recently, there has been great focus on adverse effects of herbal drugs in the medical literature; however, there have been few studies on the use of herbal drugs in pregnancy [13, 14].

*Tropaeolum majus* is a species with potential to be used in herbal drugs. It has proven efficacy as antihypertensive and diuretic [3–6] and it has been used abundantly in Brazil as food supplement rich in vitamin C and minerals. Some studies showed a relative safety in its use [3, 8, 9]; however, to the present, there are no data that clearly indicate the safety of its use during pregnancy. In this study, we show for the first time that the hydroethanolic extract obtained from the leaves of* Tropaeolum majus* can interfere with the concentration of DHEA and estradiol and reduces the possibility of proper embryo implantation in early stages of pregnancy.

Proper embryo implantation is a critical factor determining the success of a pregnancy, and this process depends on both the embryo and the endometrium, which have to develop in a highly coordinated manner. In a successful implantation, the blastocyst attaches to the endometrial epithelial cells and the trophoblastic cells invade through this layer until they reach the endometrial stroma, thus firmly anchoring the embryo into the uterine wall. At the time of implantation, the endometrium undergoes the process of decidualization and becomes morphologically and functionally distinct from their former state [15]. This decidua is thought to provide the blastocyst with factors required for further embryo development. Therefore, defects in decidualization lead to incomplete embryo implantation, which results in pregnancy loss. Recent data showed that aberrant glucose metabolism, specifically in the endometrial stroma, leads to inefficient decidualization and, thus, may be an important underlying cause of incomplete embryo implantation and, ultimately, miscarriages. Frolova et al. [16] revealed that the elevated levels of DHEA cause a strong increase in implantation failure by interfering with proper growth, development, and differentiation of the endometrium into a decidua. This inhibitory action, at least in part, can occur because of DHEA's inhibition of glucose-6-phosphate dehydrogenase (G6PD) and prevention of glucose flux through the pentose phosphate pathway (PPP). In the same way, Luchetti et al. [17] showed that the treatment with DHEA induces embryo resorption in early pregnant BALB/c mice. This fact may be related to the abolished progesterone-induced blocking factor (PIBF) expression and diminished IL-6 levels and increased IL-2 concentration.

Another important aspect to be considered refers to problems with the differentiation of the uterus to the receptive state in response to the ovarian hormones estrogen and progesterone. Normally, the “window” of uterine receptivity lasts for a limited time and estrogen levels within a very narrow range determine the duration of the window of uterine receptivity. Although estrogen at different physiological concentrations can initiate implantation, the window of uterine receptivity remains open for an extended period at lower estrogen levels but rapidly closes at higher levels [18]. Furthermore, the uterine refractoriness that follows the receptive state at high estrogen levels may be accompanied by aberrant uterine expression of implantation-related genes [19]. So, considering these aspects, we found an important reduction of embryo implantation in the female Wistar rats treated with HETM. This statement can be supported by the fact that HETM administration increased levels of DHEA and estradiol, which may lead to inefficient decidualization and, thus, may be an important underlying cause of inappropriate blastocyst acceptance and implantation.

On the other hand, when there is evidence that HETM can induce hypotensive and diuretic effect by inhibiting ACE [5, 6], we can only ask one question: is there any relationship between ACE inhibition and preimplantation losses observed? Currently, it is known that the ovarian renin-angiotensin system is implicated in a wide variety of effects in the adult human ovary, including steroidogenesis, ion fluxes, follicular growth and maturation, ovulation, angiogenesis, neovascularization, and apoptosis [20]. Most notably, local actions of angiotensin II may be directly involved in embryo implantation and uterine blood flow control during pregnancy [21]. Furthermore, it is known that ACE inhibitors cause disturbances in the ovarian rennin-angiotensin system (RAS). These changes include reproductive disorders as polycystic ovary syndrome, ovarian hyperstimulation, difficulty in embryo implantation, and ectopic pregnancy [22]. Considering the above, the possibility that the ACE inhibitor effect of* Tropaeolum majus* may in any way interfere with the humoral and hormonal imbalance and may affect zygote and early embryo transport cannot be excluded. Further studies should be conducted in order to evaluate the real participation of ACE inhibition in the embryo implantation or during the period of organogenesis.

## 5. Conclusion

In conclusion, the study showed that the HETM presents compounds responsible for increasing levels of DHEA and estradiol and may induce difficulty of implanting the embryo in early stages of pregnancy. Despite the evidence that the ACE inhibitory activity of HETM may contribute to this effect, this does not exclude that other ways operating in an integrated manner or other molecular mechanisms may be involved in the disturbance. Further, the data reported here collaborates with preclinical investigations of this species and contributes significantly to the safety aspects of using this natural product when trying to get pregnant or during pregnancy.

## Figures and Tables

**Table 1:** Maternal variables after treatment with HETM (3, 30, and 300 mg/kg) or control (aqueous solution) from the 1st to the 7th day of pregnancy.

Parameters	Experimental groups			
	Control	HETM 3	HETM 30	HETM 300
Parturient dams	15	11	13	15
Body weights (g)	261 ± 4.25	259 ± 4.81	265 ± 4.84	258 ± 3.84
Absolute organs weights				
Liver (g)	10.9 ± 0.21	9.51 ± 0.27	10.00 ± 0.18	9.77 ± 0.30
Kidneys (g)	0.81 ± 0.01	0.79 ± 0.02	0.78 ± 0.01	0.80 ± 0.02
Adrenals (mg)	19.40 ± 1.18	18.55 ± 1.42	16.69 ± 1.15	18.47 ± 1.18
Ovaries (mg)	27.00 ± 0.93	29.09 ± 2.44	27.08 ± 1.12	27.60 ± 1.36
Uterus (mg)	731.5 ± 44.1	665.9 ± 63.1	707.1 ± 56.2	663.1 ± 44.0
Relative organs weights				
Liver (%)	3.86 ± 0.05	3.66 ± 0.09	3.77 ± 0.06	3.78 ± 0.09
Kidneys (%)	0.31 ± 0.01	0.30 ± 0.01	0.29 ± 0.01	0.31 ± 0.01
Adrenals (%)	0.01 ± 0.01	0.01 ± 0.01	0.01 ± 0.01	0.01 ± 0.01
Ovaries (%)	0.01 ± 0.01	0.01 ± 0.01	0.01 ± 0.01	0.01 ± 0.01
Uterus (%)	0.28 ± 0.01	0.25 ± 0.02	0.26 ± 0.02	0.25 ± 0.01

Values are expressed as mean ± S. E. M. using Kruskal-Wallis test followed by Mann-Whitney test.
